# Vesicular Stomatitis Virus–Based Vaccines against Lassa and Ebola Viruses

**DOI:** 10.3201/eid2102.141649

**Published:** 2015-02

**Authors:** Andrea Marzi, Friederike Feldmann, Thomas W. Geisbert, Heinz Feldmann, David Safronetz

**Affiliations:** National Institutes of Health, Hamilton, Montana, USA (A. Marzi, F. Feldmann, H. Feldmann, D. Safronetz);; University of Texas Medical Branch, Galveston Texas, USA (T.W. Geisbert)

**Keywords:** vesicular stomatitis virus, VSV, viruses, pre-existing immunity, viral hemorrhagic fever, vaccines, virus-based vaccines, Lassa virus, Ebola virus, sequential vaccination, nonhuman primates, West Africa

## Abstract

We demonstrated that previous vaccination with a vesicular stomatitis virus (VSV)–based Lassa virus vaccine does not alter protective efficacy of subsequent vaccination with a VSV-based Ebola virus vaccine. These findings demonstrate the utility of VSV-based vaccines against divergent viral pathogens, even when preexisting immunity to the vaccine vector is present.

Viral hemorrhagic fevers (VHFs) are caused by a group of genetically distinct zoonotic viruses, which include ≥4 virus families (*Filoviridae*, *Arenaviridae*, *Bunyaviridae*, and *Flaviviridae*). Because of major and often highly publicized outbreaks, the most recognized VHF agents are Ebola virus (EBOV), Marburg virus (MARV), and Lassa virus (LASV). However, there are many other prominent etiologic agents of VHFs that (when infection numbers are combined) result in hundreds of thousands of infections annually, which cause a major burden to public health care systems worldwide (*1*).

Illness and death associated with these pathogens, combined with the threat of intentional release, has led to intensive research efforts to develop rapid-acting, safe, and effective medical countermeasures to control the spread of VHFs. Because of nonspecific clinical onset; rapid progression to severe disease; uncertain pathophysiology of disease; and high viral loads in blood, secretions, or excretions of affected patients that can result in human-to-human transmission, the ideal countermeasure remains prophylactic vaccination. Several vaccine platforms have shown efficacy against individual VHF pathogens, many of which are based on similar platforms (*1*). Among the most successful VHF vaccine candidates are recombinant, replication-competent vesicular stomatitis virus (VSV)–based vectors, which in animal models have proven highly effective in preventing lethal disease after challenge with a variety of high-consequence viral pathogens, including, but not limited to, EBOV, MARV, and LASV, as well as Andes (ANDV) and Nipah viruses (*2*–*5*).

Although protective efficacy of individual vaccination in challenge experiments is evident, few studies have addressed the effect of sequential, long-term, vaccination strategies with 1 vaccine platform against multiple VHFs, including the VSV-based strategy. One study showed that a single vaccination, using a vaccine made from multiple VSV filovirus vaccines, affords protection against an otherwise lethal challenge with distinct EBOV species and a MARV isolate (*6*). In addition, multiple consecutive vaccinations in a short period (≤14 days) with filovirus-specific VSV vaccines have been shown to elicit protective, possibly cross-reactive, immune responses in nonhuman primates (NHPs) (*6*,*7*). However, it remains unclear whether sequential vaccinations within a population that has mounted a complete humoral immune response (≥90 days) after an initial vaccination with a VSV-based vaccine would elicit a robust and protective immune response after vaccination with a second VSV-based vaccine.

Although this question was previously overlooked because of the restricted geographic distribution of many etiologic agents of VHF for which VSV-based vaccines have been tested, the emergence of EBOV in countries in West Africa to which LASV is endemic has heightened concerns of use and efficacy of 1 vaccine platform against multiple agents of VHF (*8*). The purpose of this study was to determine if previous vaccination with a VSV-based LASV vaccine would reduce the efficacy of subsequent vaccination with the VSV-based EBOV vaccine (Figure).

**Figure:**
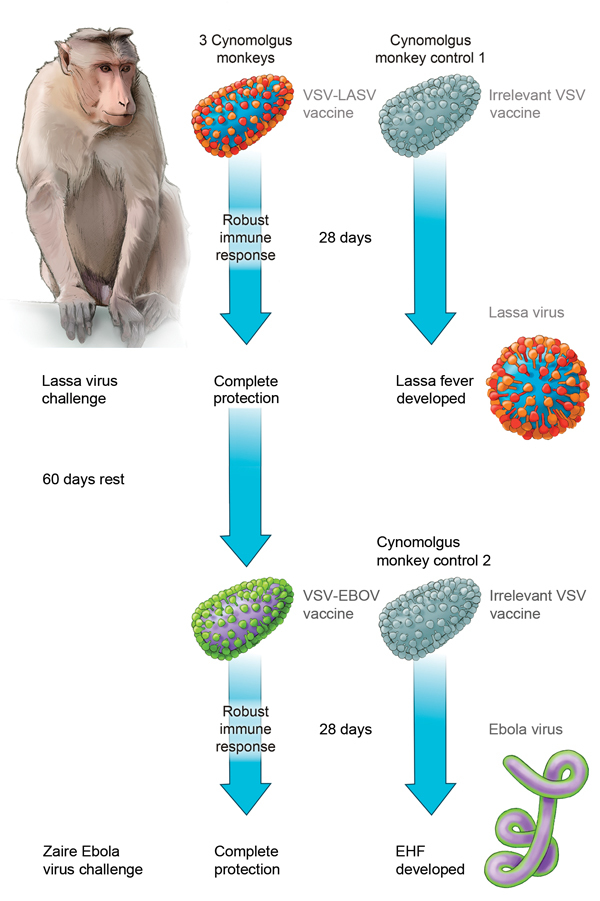
Effect of sequential vaccination with recombinant vesicular stomatitis virus (VSV)–based vaccines on protective efficacy afforded by each vaccine in nonhuman primates. Vaccination with a VSV-based Lassa virus vaccine encoding the Lassa virus glycoproteins provides complete and possibly sterile immunity against a lethal Lassa virus (LASV) challenge. Approximately 90 days after receiving the initial VSV–Lassa vaccine, animals were vaccinated with a VSV-based Ebola virus (EBOV) vaccine. Although we observed a robust VSV-specific immune response, the VSV–Ebola virus vaccine provided complete and possibly sterile immunity against a lethal Zaire Ebola virus challenge. EHF, Ebola hemorrhagic fever.

## The Study

This study was conducted in accordance with a protocol approved by an Institutional Animal Care and Use Committee of the National Institutes of Health. All laboratory work with potentially infectious materials was conducted in a Biosafety Level facility at the Rocky Mountain Laboratories (Division of Intramural Research/National Institute of Allergy and Infectious Diseases/National Institutes of Health).

Three cynomologus macaques were vaccinated with 1 dose of 10^7^ PFU of VSVΔG/LASVGPC, a live-attenuated, recombinant viral vaccine in which the VSV surface glycoprotein has been replaced with those of LASV, by intramuscular injection as described (*4*). Another age-matched control animal was vaccinated with an irrelevant VSV-based vaccine (VSVΔG/ANDVGPC) (*5*). At 28 days postvaccination, the 4 NHPs were challenged with a lethal dose of LASV (10^4^ 50% tissue culture infectious doses [TCID_50_s]) (*9*). The control animal showed signs of Lassa fever 7–10 days postinoculation and was euthanized 13 days postchallenge because of severity of disease. Classic indicators of Lassa fever, including decreased total protein and albumin; increased serum levels of alanine aminotransferase, aspartate aminotransferase, amylase, blood urea nitrogen, and alkaline phosphatase; and hematologic abnormalities, including thrombocytopenia and lymphopenia, were apparent in this animal.

Virus isolation conducted for select tissue samples showed LASV titers of 5–7 log_10_ TCID_50_/g of tissue; blood samples collected on day 10 and at the time of euthanasia (5 and 6.25 log_10_ TCID_50_/mL, respectively) showed viremia. In contrast, the 3 animals vaccinated with VSVΔG/LASVGPC resisted lethal LASV challenge and did not demonstrate any clinical signs of disease or any hematologic or biochemical indicators of LASV infection. At no point in the study was virus found in blood samples collected regularly from these 3 animals, even when tested by sensitive reverse transcription PCRs. An ELISA with serum samples collected 45 days postchallenge demonstrated equivocal antibody titers (100) against a recombinant LASV nucleocapsid protein in 1 NHP. The other 2 animals did not show seroconversion, which suggested that vaccination caused nearly sterile immunity against LASV (Table).

**Table:** Serologic responses in 3 nonhuman primates sequentially vaccinated with 2 VSV-based VHF vaccines*

Nonhuman primate no.	Lassa virus challenge study	Ebola virus challenge study

Prechallenge	Postchallenge			Prechallenge	Postchallenge		
LASV GPC	LASV GPC	LASV NP	VSV	EBOV GP	EBOV GP	EBOV VP40	VSV
1	ND	ND	100	25,600	6,400	25,600	1,600	102,400
2	ND	ND	<100	25,600	6,400	25,600	6,400	102,400
3	ND	ND	<100	25,600	6,400	25,600	6,400	102,400

*VSV, vesicular stomatitis virus; VHF, viral hemorrhagic fever; LASV, Lassa virus; GPC, glycoprotein precursor; NP, nucleocapsid protein; EBOV, Ebola virus; GP, glycoprotein; VP40 viral protein 40; ND, not determined. Titers are indicated as reciprocal endpoint dilutions from ELISAs for recombinant antigens (LASV NP, EBOV GP, and VP40), or whole virus preparations (VSV).

Approximately 90 days after the original vaccination with VSVΔG/LASVGPC, the 3 NHPs were vaccinated with a single dose of 10^7^ PFU of VSVΔG/EBOVGP by intramuscular injection as described (*3*). An additional NHP was vaccinated with a control vaccine as outlined above and served as the inoculation control. At the time of vaccination, the 3 macaques had a robust VSV-specific antibody response with titers of 25,600, as determined by a whole virus ELISA (Table). Despite this finding, the 3 animals that received the VSV-based EBOV vaccine mounted an efficient response to the EBOV glycoprotein (Table) and were completely protected when challenged 28 days later with a lethal dose of EBOV (10^3^ PFU) (*10*). Postchallenge, the 3 NHPs did not show any clinical signs of disease. Hematologic and serum biochemistry values remained constant throughout the study, and virus was not found in blood samples collected regularly and tested by using real-time reverse transcription PCR.

In contrast, severe EBOV hemorrhagic fever developed in the control animal, which was characterized by increased serum concentrations of alkaline phosphatase, aspartate aminotransferase and alanine aminotransferase; thrombocytopenia; and viremia (≥7 log_10_ TCID_50_/mL whole blood) beginning 3–6 days postchallenge. This animal was euthanized 7 days postchallenge, and titration of selected tissue samples showed EBOV titers of >9 log_10_ TCID_50_/g tissue. An ELISA conducted at the conclusion of the study (42 days post–EBOV challenge) showed increased antibody responses to VSV (titers =102,400) and seroconversion to the EBOV viral protein 40 antigen (titers 1,600–6,400) in the 3 surviving NHPs (Table), which is consistent with published results (*10*).

## Conclusions

Because of the remote locations where VHF agents are present, an overall shortage of health care professionals and clinics in these locations, and mobility of human populations, any vaccine against these pathogens would ideally need to elicit a protective immune response after a single vaccination. For this reason, replication-competent viral vectors are considered leading VHF vaccine candidates.

As the VSVΔG/EBOVGP vaccine heads toward clinical trials, it is necessary to clarify the potential limitations of using the VSV platform against multiple VHF agents. A major drawback for many viral vector platforms is preexisting immunity against the vector itself, which can decrease or nullify the essential protective immune response, which results in vaccine failure. Design of the VSV-based vaccines, which encode and express glycoproteins from various pathogens without its own glycoprotein (*11*,*12*), suggest that preexisting immunity would not influence protective efficacy of individual vaccinations (*12*). However, until now, this possibility has not been examined in disease models. Results of this study demonstrate that multiple VSV vaccines can be used in a population without any deleterious effect on overall protective efficacy.

## References

[1] Falzarano D, Feldmann H. Vaccines for viral hemorrhagic fevers-progress and shortcomings. Curr Opin Virol. 2013;3:343–51. 10.1016/j.coviro.2013.04.007 PMC3743920 23773330

[2] DeBuysscher BL, Scott D, Marzi A, Prescott J, Feldmann H. Single-dose live-attenuated Nipah virus vaccines confer complete protection by eliciting antibodies directed against surface glycoproteins. Vaccine. 2014;32:2637–44. 10.1016/j.vaccine.2014.02.087 24631094 PMC4829066

[3] Jones SM, Feldmann H, Stroher U, Geisbert JB, Fernando L, Grolla A, Live attenuated recombinant vaccine protects nonhuman primates against Ebola and Marburg viruses. Nat Med. 2005;11:786–90. 10.1038/nm1258 15937495

[4] Geisbert TW, Jones S, Fritz EA, Shurtleff AC, Geisbert JB, Liebscher R, Development of a new vaccine for the prevention of Lassa fever. PLoS Med. 2005;2:e183. 10.1371/journal.pmed.0020183 15971954 PMC1160587

[5] Brown KS, Safronetz D, Marzi A, Ebihara H, Feldmann H. Vesicular stomatitis virus-based vaccine protects hamsters against lethal challenge with Andes virus. J Virol. 2011;85:12781–91. 10.1128/JVI.00794-11 21917979 PMC3209372

[6] Geisbert TW, Geisbert JB, Leung A, Daddario-DiCaprio KM, Hensley LE, Groll A, Single-injection vaccine protects nonhuman primates against infection with Marburg virus and three species of Ebola virus. J Virol. 2009;83:7296–304. 10.1128/JVI.00561-09 19386702 PMC2704787

[7] Mire CE, Geisbert JB, Marzi A, Agans KN, Feldmann H, Geisbert TW. Vesicular stomatitis virus-based vaccines protect nonhuman primates against Bundibugyo ebolavirus. PLoS Negl Trop Dis. 2013;7:e2600. 10.1371/journal.pntd.0002600 24367715 PMC3868506

[8] WHO Ebola Response Team. Ebola virus disease in West Africa—the first 9 months of the epidemic and forward projections. N Engl J Med. 2014;371:1481–95. 10.1056/NEJMoa1411100 25244186 PMC4235004

[9] Safronetz D, Strong JE, Feldmann F, Haddock E, Sogoba N, Brining D, A recently isolated Lassa virus from Mali demonstrates atypical clinical disease manifestations and decreased virulence in cynomolgus macaques. J Infect Dis. 2013;207:1316–27. 10.1093/infdis/jit004 23303805 PMC3603532

[10] Marzi A, Engelmann F, Feldmann F, Haberthur K, Shupert WL, Brining D, Antibodies are necessary for rVSV/ZEBOV-GP-mediated protection against lethal Ebola virus challenge in nonhuman primates. Proc Natl Acad Sci U S A. 2013;110:1893–8. 10.1073/pnas.1209591110 23319647 PMC3562844

[11] Garbutt M, Liebscher R, Wahl-Jensen V, Jones S, Möller P, Wagner R, Properties of replication-competent vesicular stomatitis virus vectors expressing glycoproteins of filoviruses and arenaviruses. J Virol. 2004;78:5458–65. 10.1128/JVI.78.10.5458-5465.2004 15113924 PMC400370

[12] Rose NF, Roberts A, Buonocore L, Rose JK. Glycoprotein exchange vectors based on vesicular stomatitis virus allow effective boosting and generation of neutralizing antibodies to a primary isolate of human immunodeficiency virus type 1. J Virol. 2000;74:10903–10. 10.1128/JVI.74.23.10903-10910.2000 11069984 PMC113169

